# Supplementary material for: End Sequence Analysis Toolkit (ESAT) expands the extractable information from single-cell RNA-seq data
Source: Genome Res. 2016 Oct;26(10):1397–410. doi: 10.1101/gr.207902.116 (PMC5052061; doi:10.1101/gr.207902.116)
Supplement: Supplemental Material [file supp_gr.207902.116_Supp_Materials.tar.gz › ESAT-esat_v0.1/doc/html/classbroad_1_1core_1_1annotation_1_1_g_f_f-members.html]

ESAT: Member List


ESAT


- Main Page
- Related Pages
- Packages
- Classes
- Files

- Class List
- Class Index
- Class Hierarchy
- Class Members

All Classes Namespaces Files Functions Variables Enumerator Pages

- broad
- core
- annotation
- GFF

broad.core.annotation.GFF Member List

This is the complete list of members for broad.core.annotation.GFF, including all inherited members.

|  |  |  |
| --- | --- | --- |
| addAttribute(String attributeName, String attributeValue) | broad.core.annotation.GFF |  |
| addAttributeValues(String attributeName, List< String > values) | broad.core.annotation.GFF |  |
| addBlock(String name, int start, int end) | broad.core.annotation.BasicGenomicAnnotation |  |
| addExtraScore(double score) | broad.core.annotation.BasicLightweightAnnotation |  |
| almostEqual(LightweightGenomicAnnotation other, int fudge) | broad.core.annotation.BasicLightweightAnnotation |  |
| BasicGenomicAnnotation() | broad.core.annotation.BasicGenomicAnnotation | protected |
| BasicGenomicAnnotation(String name) | broad.core.annotation.BasicGenomicAnnotation |  |
| BasicGenomicAnnotation(String name, String chr, int start, int end) | broad.core.annotation.BasicGenomicAnnotation |  |
| BasicGenomicAnnotation(String name, String chr, int start, int end, Strand strand) | broad.core.annotation.BasicGenomicAnnotation |  |
| BasicGenomicAnnotation(LightweightGenomicAnnotation ga) | broad.core.annotation.BasicGenomicAnnotation |  |
| BasicGenomicAnnotation(Annotation ga) | broad.core.annotation.BasicGenomicAnnotation |  |
| BasicGenomicAnnotation(GenomicAnnotation ga) | broad.core.annotation.BasicGenomicAnnotation |  |
| BasicGenomicAnnotation(String[] info) | broad.core.annotation.BasicGenomicAnnotation |  |
| BasicLightweightAnnotation() | broad.core.annotation.BasicLightweightAnnotation |  |
| BasicLightweightAnnotation(String chr, int start, int end) | broad.core.annotation.BasicLightweightAnnotation |  |
| BasicLightweightAnnotation(String chr, int start, int end, String orientation) | broad.core.annotation.BasicLightweightAnnotation |  |
| BasicLightweightAnnotation(String chr, int start, int end, Strand orientation, String name) | broad.core.annotation.BasicLightweightAnnotation |  |
| BasicLightweightAnnotation(String chr, int start, int end, String orientation, double Scr) | broad.core.annotation.BasicLightweightAnnotation |  |
| BasicLightweightAnnotation(String chr, String start, String end) | broad.core.annotation.BasicLightweightAnnotation |  |
| BasicLightweightAnnotation(LightweightGenomicAnnotation annotation) | broad.core.annotation.BasicLightweightAnnotation |  |
| BasicLightweightAnnotation(Annotation annotation) | broad.core.annotation.BasicLightweightAnnotation |  |
| clearAttribute(String attributeName) | broad.core.annotation.GFF |  |
| compareTo(GenomicAnnotation arg0) | broad.core.annotation.BasicGenomicAnnotation |  |
| broad::core::annotation::BasicLightweightAnnotation.compareTo(LightweightGenomicAnnotation b) | broad.core.annotation.BasicLightweightAnnotation |  |
| compareToAnnotation(Annotation b) | umms.core.annotation.Annotation |  |
| complement() | umms.core.annotation.Annotation |  |
| contains(LightweightGenomicAnnotation other) | broad.core.annotation.BasicLightweightAnnotation |  |
| umms::core::annotation::Annotation.contains(Annotation other) | umms.core.annotation.Annotation |  |
| copy() | umms.core.annotation.Annotation |  |
| createFromUCSC(String ucsc) | broad.core.annotation.BasicLightweightAnnotation | static |
| disect(Annotation a) | broad.core.annotation.BasicGenomicAnnotation |  |
| disect(List<?extends Annotation > disectors) | broad.core.annotation.BasicGenomicAnnotation |  |
| equals(Annotation other) | umms.core.annotation.Annotation |  |
| equals(Annotation other, boolean useOrientation) | umms.core.annotation.Annotation |  |
| expand(int deltaStart, int deltaEnd) | umms.core.annotation.Annotation |  |
| fullyContains(Annotation annotation) | umms.core.annotation.Annotation |  |
| getAttributes() | broad.core.annotation.GFF |  |
| broad::core::annotation::BasicLightweightAnnotation.getBlocks() | umms.core.annotation.Annotation |  |
| broad::core::annotation::BasicLightweightAnnotation.getBlocks(boolean oriented) | umms.core.annotation.Annotation |  |
| broad::core::annotation::GenomicAnnotation.getBlocks() | broad.core.annotation.GenomicAnnotation |  |
| getChr() | umms.core.annotation.Annotation |  |
| getChromosome() | broad.core.annotation.BasicLightweightAnnotation |  |
| getChromosomeString() | broad.core.annotation.BasicLightweightAnnotation |  |
| getDistanceTo(LightweightGenomicAnnotation other) | broad.core.annotation.BasicLightweightAnnotation |  |
| umms::core::annotation::Annotation.getDistanceTo(Annotation other) | umms.core.annotation.Annotation |  |
| getEnd() | broad.core.annotation.LightweightGenomicAnnotation |  |
| getExtraScore(int i) | broad.core.annotation.BasicLightweightAnnotation |  |
| getExtraScores() | broad.core.annotation.BasicLightweightAnnotation |  |
| getFeature() | broad.core.annotation.GFF |  |
| getFirstValue(String attributeName) | broad.core.annotation.GFF |  |
| getFivePrimeBases() | broad.core.annotation.BasicGenomicAnnotation |  |
| getFrame() | broad.core.annotation.GFF |  |
| getFullInfoString() | umms.core.annotation.Annotation |  |
| getGroup() | broad.core.annotation.GFF |  |
| getGTFAttr(String rawFields, String attrName) | broad.core.annotation.GFF | static |
| getGTFAttr(String[] attributeArray, String attrName) | broad.core.annotation.GFF | static |
| getId() | broad.core.annotation.BasicGenomicAnnotation |  |
| getLength() | broad.core.annotation.BasicGenomicAnnotation |  |
| getLengthOnReference() | umms.core.annotation.Annotation |  |
| broad::core::annotation::BasicLightweightAnnotation.getLocationString() | broad.core.annotation.BasicLightweightAnnotation |  |
| broad::core::annotation::GenomicAnnotation.getLocationString() | broad.core.annotation.GenomicAnnotation |  |
| getMiddle() | broad.core.annotation.BasicLightweightAnnotation |  |
| getMidpoint() | umms.core.annotation.Annotation |  |
| broad::core::annotation::BasicLightweightAnnotation.getName() | broad.core.annotation.LightweightGenomicAnnotation |  |
| broad::core::annotation::GenomicAnnotation.broad::core::annotation::Feature.getName() | broad.core.annotation.Feature |  |
| broad::core::annotation::BasicLightweightAnnotation.getOrientation() | broad.core.annotation.LightweightGenomicAnnotation |  |
| broad::core::annotation::GenomicAnnotation.getOrientation() | broad.core.annotation.GenomicAnnotation |  |
| getOrientedEnd() | broad.core.annotation.BasicGenomicAnnotation |  |
| getOrientedStart() | broad.core.annotation.BasicGenomicAnnotation |  |
| getOverlap(LightweightGenomicAnnotation other) | broad.core.annotation.BasicLightweightAnnotation |  |
| umms::core::annotation::Annotation.getOverlap(Annotation other) | umms.core.annotation.Annotation |  |
| getPositionAtReferenceCoordinate(int referenceCoordinate) | umms.core.annotation.Annotation |  |
| getPositionAtReferenceCoordinate(int referenceCoordinate, boolean ignoreOrientation) | umms.core.annotation.Annotation |  |
| getReferenceCoordinateAtPosition(int positionInAnnotation) | umms.core.annotation.Annotation |  |
| getReferenceCoordinateAtPosition(int positionInAnnotation, boolean ignoreOrientation) | umms.core.annotation.Annotation |  |
| getReferenceName() | umms.core.annotation.Annotation |  |
| getSAMEnd() | umms.core.annotation.Annotation |  |
| getSAMStart() | umms.core.annotation.Annotation |  |
| broad::core::annotation::BasicLightweightAnnotation.getScore() | broad.core.annotation.BasicLightweightAnnotation |  |
| broad::core::annotation::GenomicAnnotation.broad::core::annotation::Feature.getScore() | broad.core.annotation.Feature |  |
| getSequence() | broad.core.annotation.BasicGenomicAnnotation |  |
| getSequenceName() | broad.core.annotation.GFF |  |
| getSize() | umms.core.annotation.Annotation |  |
| getSource() | broad.core.annotation.GFF |  |
| getSpliceConnections() | umms.core.annotation.Annotation |  |
| getStart() | broad.core.annotation.LightweightGenomicAnnotation |  |
| getStrand() | broad.core.annotation.GFF |  |
| getThreePrimeBases() | broad.core.annotation.BasicGenomicAnnotation |  |
| getValues(String attributeName) | broad.core.annotation.GFF |  |
| GFF() | broad.core.annotation.GFF |  |
| GFF(String name) | broad.core.annotation.GFF |  |
| GFF(LightweightGenomicAnnotation annotation) | broad.core.annotation.GFF |  |
| GFF(Annotation annotation) | broad.core.annotation.GFF |  |
| GFF(GFF gff) | broad.core.annotation.GFF |  |
| GFF(String[] rawFields) | broad.core.annotation.GFF |  |
| hashCode() | broad.core.annotation.BasicLightweightAnnotation |  |
| hasOrientation() | umms.core.annotation.Annotation |  |
| inReversedOrientation() | broad.core.annotation.BasicLightweightAnnotation |  |
| intersect(LightweightGenomicAnnotation other) | broad.core.annotation.BasicLightweightAnnotation |  |
| intersect(Collection<?extends Annotation > annotations) | broad.core.annotation.BasicLightweightAnnotation |  |
| broad::core::annotation::LightweightGenomicAnnotation.intersect(Annotation other) | umms.core.annotation.Annotation |  |
| broad::core::annotation::LightweightGenomicAnnotation.intersect(List<?extends Annotation > annotations) | umms.core.annotation.Annotation |  |
| isFlankedBy(TwoSubjectAnnotation twoSubjectAnnotation, int buffer) | broad.core.annotation.BasicGenomicAnnotation |  |
| isNegativeStrand() | umms.core.annotation.Annotation |  |
| isUnoriented() | umms.core.annotation.Annotation |  |
| lastIdxWhereListSearchBroke() | broad.core.annotation.BasicGenomicAnnotation |  |
| length() | broad.core.annotation.BasicLightweightAnnotation |  |
| mayHaveBlocks() | broad.core.annotation.BasicGenomicAnnotation |  |
| mergeAllOverlappers(TreeSet< BasicLightweightAnnotation > regions) | broad.core.annotation.BasicLightweightAnnotation | static |
| minus(Annotation other) | umms.core.annotation.Annotation |  |
| minus(Collection<?extends Annotation > others) | umms.core.annotation.Annotation |  |
| moveToCoordinate(int coordinateInReference) | umms.core.annotation.Annotation |  |
| numBlocks() | umms.core.annotation.Annotation |  |
| overlaps(Collection<?extends Annotation > others, int buffer) | broad.core.annotation.BasicLightweightAnnotation |  |
| overlaps(Collection<?extends Annotation > others) | broad.core.annotation.BasicLightweightAnnotation |  |
| broad::core::annotation::LightweightGenomicAnnotation.overlaps(Annotation other, int buffer) | umms.core.annotation.Annotation |  |
| broad::core::annotation::LightweightGenomicAnnotation.overlaps(Annotation other) | umms.core.annotation.Annotation |  |
| broad::core::annotation::LightweightGenomicAnnotation.overlaps(Annotation other, int buffer, boolean considerOrientation) | umms.core.annotation.Annotation |  |
| broad::core::annotation::LightweightGenomicAnnotation.overlaps(Annotation other, boolean considerOrientation) | umms.core.annotation.Annotation |  |
| overlapsStranded(Annotation other) | umms.core.annotation.Annotation |  |
| reduceToDifference(GenomicAnnotation other) | broad.core.annotation.BasicGenomicAnnotation |  |
| removeExtraScores() | broad.core.annotation.BasicLightweightAnnotation |  |
| setAttributes(String rawAttributeData) | broad.core.annotation.GFF |  |
| setBoundariesFromAnnoations(List<?extends GenomicAnnotation > annotations) | broad.core.annotation.BasicLightweightAnnotation |  |
| setChromosome(String chr) | broad.core.annotation.BasicLightweightAnnotation |  |
| setEnd(int end) | broad.core.annotation.LightweightGenomicAnnotation |  |
| setFeature(String feature) | broad.core.annotation.GFF |  |
| setFivePrimeBuffer(int bufferSize) | broad.core.annotation.BasicGenomicAnnotation |  |
| setFrame(int frame) | broad.core.annotation.GFF |  |
| setGroup(String group) | broad.core.annotation.GFF |  |
| setId(String id) | broad.core.annotation.BasicGenomicAnnotation |  |
| setName(String name) | broad.core.annotation.LightweightGenomicAnnotation |  |
| setOrientation(boolean orientation) | broad.core.annotation.BasicGenomicAnnotation |  |
| broad::core::annotation::BasicLightweightAnnotation.setOrientation(String orientation) | broad.core.annotation.BasicLightweightAnnotation |  |
| broad::core::annotation::BasicLightweightAnnotation.umms::core::annotation::Annotation.setOrientation(char orientation) | umms.core.annotation.Annotation |  |
| broad::core::annotation::BasicLightweightAnnotation.umms::core::annotation::Annotation.setOrientation(Strand orientation) | umms.core.annotation.Annotation |  |
| broad::core::annotation::GenomicAnnotation.setOrientation(String orientationString) | broad.core.annotation.GenomicAnnotation |  |
| setOrientedEnd(int orientedEnd) | umms.core.annotation.Annotation |  |
| setOrientedStart(int orientedStart) | umms.core.annotation.Annotation |  |
| setReferenceName(String refName) | umms.core.annotation.Annotation |  |
| broad::core::annotation::BasicLightweightAnnotation.setReversedOrientation(boolean isInReversedOrientation) | broad.core.annotation.BasicLightweightAnnotation |  |
| broad::core::annotation::GenomicAnnotation.setReversedOrientation(boolean isInReversedOrientation) | broad.core.annotation.GenomicAnnotation |  |
| setScore(double score) | broad.core.annotation.BasicLightweightAnnotation |  |
| setSequence(Sequence sequence) | broad.core.annotation.BasicGenomicAnnotation |  |
| setSource(String source) | broad.core.annotation.GFF |  |
| setStart(String data) | broad.core.annotation.BasicLightweightAnnotation | protected |
| broad::core::annotation::LightweightGenomicAnnotation.setStart(int start) | broad.core.annotation.LightweightGenomicAnnotation |  |
| setStrand(String strand) | broad.core.annotation.GFF |  |
| setThreePrimeBuffer(int bufferSize) | broad.core.annotation.BasicGenomicAnnotation |  |
| shift(int delta) | umms.core.annotation.Annotation |  |
| size() | umms.core.annotation.Annotation |  |
| stitchList(Collection<?extends Annotation > sortedList, int maxDistanceToStitch) | broad.core.annotation.BasicLightweightAnnotation | static |
| stitchTo(LightweightGenomicAnnotation other) | broad.core.annotation.BasicLightweightAnnotation |  |
| umms::core::annotation::Annotation.stitchTo(Annotation next) | umms.core.annotation.Annotation |  |
| takeIntersection(LightweightGenomicAnnotation other) | broad.core.annotation.BasicLightweightAnnotation |  |
| takeUnion(LightweightGenomicAnnotation other) | broad.core.annotation.BasicLightweightAnnotation |  |
| toBED() | umms.core.annotation.Annotation |  |
| toBED(int r, int g, int b) | umms.core.annotation.Annotation |  |
| toBEDGraph() | umms.core.annotation.Annotation |  |
| toCigar() | umms.core.annotation.Annotation |  |
| toCufflinksString(boolean quoteValues) | broad.core.annotation.GFF |  |
| toShortBED() | umms.core.annotation.Annotation |  |
| toString() | broad.core.annotation.GFF |  |
| toString(boolean quoteValues) | broad.core.annotation.GFF |  |
| toString(boolean quoteValues, boolean useEqualSign) | broad.core.annotation.GFF |  |
| toUCSC() | broad.core.annotation.BasicLightweightAnnotation |  |
| trim(int deltaStart, int deltaEnd) | umms.core.annotation.Annotation |  |
| union(Annotation other) | umms.core.annotation.Annotation |  |


---

Generated on Fri Aug 15 2014 15:10:51 for ESAT by  

 1.8.7
